# Subjective Age, Future Care Preparation, and Their Association in Dementia Caregivers and Never-Caregivers

**DOI:** 10.1093/geroni/igaf122.221

**Published:** 2025-12-31

**Authors:** Serena Sabatini, Emily Mroz, Shelbie Turner, Fiona Rupprecht, Victoria Tischler, Blossom Stephan

**Affiliations:** University of Barcelona, Barcelona, Catalonia, Spain; Emory University, Atlanta, Georgia, United States; Weill Cornell Medicine, New York, New York, United States; University of Vienna, Vienna, Wien, Austria; University of Surrey School of Psychology, Guildford, England, United Kingdom; Curtin EnAble Institute, Perth, Western Australia, Australia

## Abstract

Subjective age refers to the age an individual feels like, which can differ from their chronological age. Future care preparation refers to the process of sharing preferences regarding future care with health-care professionals, family, or friends. Engagement in future care preparation is important as it is related to positive psychological outcomes (i.e., lower anxiety and depression), and it limits interpersonal and economic challenges. This study examined differences in subjective age and future care preparation in dementia caregivers vs. never-caregivers in the UK, and investigated the moderating role of caregiving status in the association of subjective age with future care preparation. An online survey was conducted with 259 informal dementia caregivers (Mean age=62.83; SD = 9.24) and 1699 never-caregivers (Mean age=67.79 years; SD = 8.05 years). Unadjusted and adjusted (for age, sex, education, marital status, working status, physical and mental health) regression models were estimated. Never-caregivers felt 4% younger than dementia caregivers (p<.001). Although both dementia caregivers and never-caregivers reported low future care preparation, being a dementia caregiver was significantly associated with more future care preparation (Mean=13.65) compared to never-caregivers (Mean=12.67). In the overall sample a higher subjective age was associated with greater future care preparation (Adjusted unstandardized regression coefficient=-1.71; 95%CI: -3.01, -0.39). Caregiving status did not moderate the association of subjective age with future care preparation. Hence, a higher subjective age is related to more future care preparation both in dementia caregivers and never-caregivers. Strategies promoting future care preparation maybe more effective if they focus on sense of ageing rather than targeting caregiver status.

